# Fast Repetition Rate Fluorometry (FRRF) Derived Phytoplankton Primary Productivity in the Bay of Bengal

**DOI:** 10.3389/fmicb.2019.01164

**Published:** 2019-05-24

**Authors:** Yuqiu Wei, Xiangwei Zhao, Jun Sun, Haijiao Liu

**Affiliations:** ^1^Institute of Marine Science and Technology, Shandong University, Qingdao, China; ^2^Research Centre for Indian Ocean Ecosystem, Tianjin University of Science and Technology, Tianjin, China; ^3^Tianjin Key Laboratory of Marine Resources and Chemistry, Tianjin University of Science and Technology, Tianjin, China

**Keywords:** phytoplankton, primary production, Bay of Bengal, photosynthetic parameters, electron transport

## Abstract

The approach of fast repetition rate fluorometry (FRRF) requires a conversion factor (Φ_e : C_/*n*_PSII_) to derive ecologically-relevant carbon uptake rates (*PP*_z,t_). However, the required Φ_e : C_/*n*_PSII_ is commonly measured by ^14^C assimilation and varies greatly across phytoplankton taxonomy and environmental conditions. Consequently, the use of FRRF to estimate gross primary productivity (*GP*_z,t_), alone or in combination with other approaches, has been restricted by both inherent conversion and procedural inconsistencies. Within this study, based on a hypothesis that the non-photochemical quenching (NPQ_NSV_) can be used as a proxy for the variability and magnitude of Φ_e : C_/*n*_PSII_, we thus proposed an independent field model coupling with the NPQ_NSV_-based Φ_e : C_/*n*_PSII_ for FRRF-derived carbon, without the need for additional Φ_e : C_/*n*_PSII_ in the Bay of Bengal (BOB). Therewith, this robust algorithm was verified by the parallel measures of electron transport rates and ^14^C-uptake *PP*_z,t_. NPQ_NSV_ is theoretically caused by the effects of excess irradiance pressure, however, it showed a light and depth-independent response on large spatial scales of the BOB. Trends observed for the maximum quantum efficiency (F_v_/F_m_), the quantum efficiency of energy conversion (${\text{F}}_{\text{q}}^{\prime }$/${\text{F}}_{\text{m}}^{\prime }$) and the efficiency of charge separation (${\text{F}}_{\text{q}}^{\prime }$/${\text{F}}_{\text{v}}^{\prime }$) were similar and representative, which displayed a relative maximum at the subsurface and were collectively limited by excess irradiance. In particular, most observed values of F_v_/F_m_ in the BOB were only about half of the values expected for nutrient replete phytoplankton. FRRF-based estimates of electron transport at PSII (ETR_RCII_) varied significantly, from 0.01 to 8.01 mol e^−^ mol RCII^−1^ s^−1^, and showed profound responses to depth and irradiance across the BOB, but fitting with the logistic model. N, P, and irradiance are key environmental drivers in explaining the broad-scale variability of photosynthetic parameters. Furthermore, taxonomic shifts and physiological changes may be better predictors of photosynthetic parameters, and facilitate the selection of better adapted species to optimize photosynthetic efficiency under any particular set of ambient light condition.

## Introduction

A convenient starting-point in marine ecosystem cycle is the photosynthesis from phytoplankton, with a possible very minor contribution of a few species of truly photosynthetic bacteria (McDermott et al., 1995). Marine phytoplankton annually fix between 30 and 50 billion metric tons of carbon, which account for ~40–50% of global carbon fixation (Raymont, 2014). On ecological and geological scales, there is profound evidence of the significance of phytoplankton photosynthesis in global biogeochemical cycling. The ability to accurately measure, monitor, and predict spatiotemporal variations of ocean primary productivity and its dynamic response to external environmental conditions is therefore crucial. Traditionally, the rates of phytoplankton primary productivity have been measured tracing the evolution of O_2_ or the assimilation of CO_2_ (Tortell, 2000), as well as using the ^14^C-method in conjunction with a simulated *in situ* incubator (Gall et al., 1999). However, these techniques have a number of well-known limitations, e.g., high labor intensity and cost associated with routine sample processing, low spatial and temporal resolution, and bottle artifacts due to exclusion of contamination. Thus, there is somewhat a need for a simple, non-intrusive and inexpensive assay for productivity estimates in both coastal and oligotrophic open water researches that adequately deals with the now constrained problems. More recently, active chlorophyll *a* fluorescence (ChlF) approaches which refer to measures of the quantum yield of linear electron transport through photosystem II (PSII), including fast repetition rate fluorometry (FRRF), can afford instantaneous estimates of gross primary productivity (*GP*_z,t_) at unprecedented high spatial and temporal resolution, avoiding the artifacts related to bottle containment (Kolber and Falkowski, 1993; Kolber et al., 1998; Smyth et al., 2004).

FRRF has been widely considered a major development for marine research in global efforts to better understand environmental regulation of *GP*_z,t_ (Suggett et al., 2009). This is, to some extent, due to FRRF-derived *GP*_z,t_ rates are typically based on the estimates of electron transfer rate at PSII (i.e., rates of charge separation, ETR_RCII_, mol e^−^ mol RCII^−1^ s^−1^), which can be converted into ecologically relevant units of carbon fixation combining with derived conversion factor. The conversion factor linking ETR_RCII_ and CO_2_ uptake rates covers two parameters, the amount of chlorophyll *a* (Chl *a*) per reaction center at functional PSII (RCII; 1/*n*_PSII_, mol chl *a* mol RCII^−1^) and the electron requirement for conversion of per inorganic carbon (Φ_e : C_, mol e^−^ mol C^−1^) (Schuback et al., 2016). On the basis of large empirical comparison of FRRF-derived ETR_RCII_ and ^14^C-uptake measurements, the conversion factor Φ_e : C_/*n*_PSII_ required to derive carbon fixation estimates from the FRRF-derived rates of ETR_RCII_ appears highly variable in response to the interacting effects of micronutrient and light availability (Zhu et al., 2017), over diurnal cycles (Schuback, 2016), and in response to changes in the composition of phytoplankton assemblages (Schuback et al., 2017). In particular, the long time in ^14^C-incubation experiments may exacerbate cumulative processes such as spectral quality of the light sources used and photodamage under excess irradiance, and then influence the absolute magnitude of derived Φ_e : C_/*n*_PSII_. Overall, the commonly captured Φ_e : C_/*n*_PSII_ in the coupling between FRRF-derived productivity rates and ^14^C-assimilation data is great plasticity and not constant given its variable response to taxonomy and ambient conditions. In future work, an effort should be made to derive accurate algorithms for extrapolating the Φ_e : C_/*n*_PSII_ from FRRF-based measurements and to simulate their regulation mechanism on physiological level.

Regulation of absorption and utilization of light energy is necessary for algae to alleviate excess excitation energy after charge separation and minimize the potential for photooxidative damage (Schreiber et al., 1986). As such, marine phytoplankton evolve to optimize photosynthetic efficiency under a range of fluctuating light conditions since the way of getting rid of excess light to achieve energy-allocation balance, which can be estimated as non-photochemical quenching (NPQ_NSV_) (Schreiber et al., 1986; Müller et al., 2001). Subsequent studies have demonstrated that the NPQ_NSV_ provides mechanistic insight into the processes decoupling photosynthetic electron transport and CO_2_-assimilation (e.g., Zehr and Kudela, 2009). Both Schuback et al. (2015, 2016) and Hughes et al. (2018) demonstrated the Φ_e : C_/*n*_PSII_ variance can be correlated to FRRF-based measurements of NPQ_NSV_, interpreted as an indication of processes consuming photosynthetically derived energy and decoupling linear electron flow from carbon uptake. Accordingly, the NPQ_NSV_ can be used as a proxy for the variance and magnitude of Φ_e : C_/*n*_PSII_ between ^14^C-uptake rates and FRRf-derived ETR_RCII_ to estimate the carbon-based rates of productivity.

Yet to our knowledge, there are no direct experimental investigates in *GP*_z,t_ of natural phytoplankton assemblages based on ChlF yields as measured by FRRF in the Bay of Bengal (BOB). We thus conducted parallel measures of FRRF-derived *GP*_z,t_ and (^14^C) carbon uptake rates (*PP*_z,t_) for the BOB. Most importantly, we presented an independent field model based on the NPQ_NSV_-proxy hypothesis, without the need for additional Φ_e : C_/*n*_PSII_ in natural phytoplankton assemblages. Despite the precursor of NPQ_NSV_-based Φ_e : C_/*n*_PSII_ derived from subarctic Pacific, to validate whether this hypothesis is possible to apply in the BOB, we subsequently compared our model with previously reported models from other marine ecosystems and synchronously measured *PP*_z,t_ dataset. On large spatial scales, this is also the first study that shows NPQ_NSV_, F_v_/F_m_, ${\text{F}}_{\text{q}}^{\prime }$/${\text{F}}_{\text{m}}^{\prime }$, ${\text{F}}_{\text{q}}^{\prime }$/${\text{F}}_{\text{v}}^{\prime }$, ETR_RCII_, FRRF-derived *GP*_z,t_ and ^14^C-uptake *PP*_z,t_ in natural phytoplankton assemblages to better understand the potential environmental responses and physiological processes accounting for their variability in the BOB.

## Methods

### Study Area and Sample Collection

**Figure 1:**
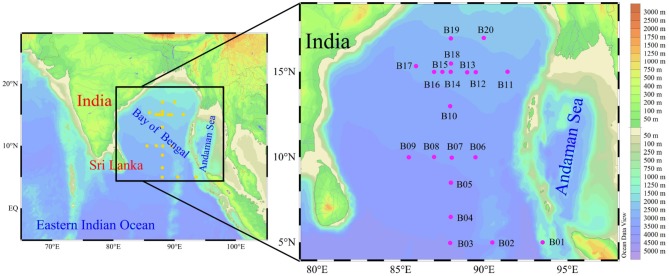
Study area and sampling locations.

Field sampling was conducted in the BOB and its adjacent shelf (5°N~20°N, 85°E~95°E) during winter 2016 (between 15th November and 18th December). Our study area covered the entire sea basin of BOB, and 20 stations (B01–B20) were investigated (Figure 1). Water samples for FRRF measurement (FastOcean, Chelsea Technologies Group, Ltd.) from the upper euphotic depth (Z_eu_, depth with 1% of surface PAR) were collected using a rosette equipped with 12L Niskin bottles (General Oceanics) and a CTD (Conductivity, Temperature and Depth; SBE 19 Plus). Samples for phytoplankton (cell size > 2 μm) were fixed with 1–2% buffered formalin and were identified under an inverted microscope (Motic BA300) following the methods outlined in Utermöhl (1958). Five hundred milliliters of seawater were filtered through 0.7 μm GF/F filters under low vacuum pressure (<0.04 MPa) to retrieve the Chl *a*. Filters were extracted in 5 mL 90% acetone for 24 h in darkness at −20°C, and the Chl *a* concentrations were determined fluorimetrically using a pre-calibrated fluorometer following Welschmeyer (1994). Hundred milliliter seawater for nitrate + nitrite + ammonium (DIN), phosphate (DIP), and silicate (DSi) was measured using a Technicon AA3 Auto-Analyzer (Bran+Luebbe) following Dai et al. (2008). Detection limit based on this approach was 0.01 μmol L^−1^. The depth profile of photosynthetically active radiation (PAR, μmol quanta m^−2^ s^−1^, 400–700 nm) was measured using an underwater PAR sensor (RBR, XRX-620). The optical extinction coefficient (including *in situ* PAR), *K*_d_ (m^−1^), was calculated as:

(1) $${\text{E}}_{\text{Z}}={\text{E}}_{\text{0}}{\;\exp}^{-(K_{\text{d}}\times \text{Z})}$$

where E_0_ is surface light intensity and E_z_ is light intensity at depth Z (m).

### FRRF-Derived Photophysiological Parameters

**Figure 2:**
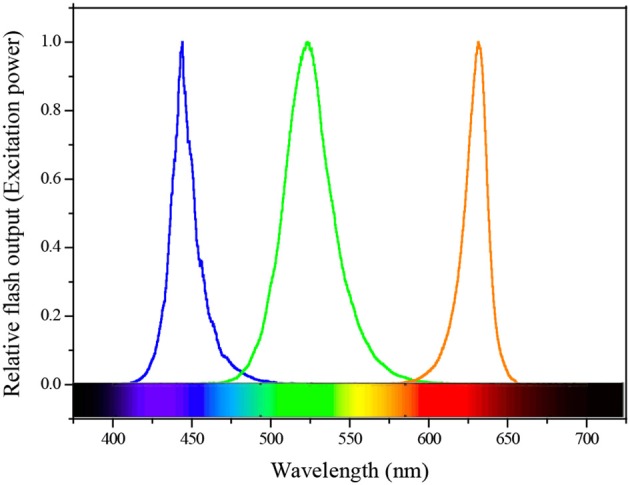
Spectral quality of the actinic flash and excitation power used for FRRF3 sensor.

All active ChlF measurements were conducted on a FastOcean FRRF3 sensor with Act2 system in the field. Water samples were kept in low light to allow the oxidation of electron transport chain (ETC) and relaxation of NPQ. A single-turnover (ST) protocol consisted of 100 flashlets (Fet, a single 1 μs excitation pulse from the LEDs within a FRRF3 sensor) with 2.0 μs Fet pitch (interval between the start of one Fet and the next). During the cruise, we measured the ST flashlet sequences continuously (2.0 μs interval) and optimized the length of each light step to allow all derived parameters to reach steady state. Excitation power was provided by LEDs at three wavelengths centered on 450 (blue), 530 (green), and 624 (orange) nm (Figure 2), and was automatically selected to saturate the observed fluorescence transients. The blue LEDs will excite Chl *a* pigment, which covers most photosynthetic algae such as diatoms and dinoflagellates etc. Cyanobacteria will have some Chl *a* in their core complexes, but do not use Chl *a* as their primary pigment to absorb light, instead they have phycobilisomes containing various phycobilin pigments, which excite at longer wavelengths ranging from green and orange/red light (McConnell et al., 2002). In ocean mixed phytoplankton communities, therefore, we simultaneously added three wavelengths to cover the broad range of absorption spectrum to improve the light absorption and generate a saturating pulse (enough light absorbed to close all RCIIs). Each sample was exposed sequentially to 12 actinic background irradiances spanning from 0 to 1,200 μmol quanta m^−2^ s^−1^ to retrieve fluorescence-light response curves, also provided at three wavelengths. The time of subsequent light adaptation is twice as long as the initial dark condition. This is because a high proportion of phytoplankton assemblages require substantially longer to adapt to the initial transition between dark and light than to adapt to small increases in photon irradiance.

ChlF yields and FRRF-derived parameters corresponding to each actinic light level were recorded from the average of all acquisitions (Acqs). F_o_ is the initial ChlF yield induced by a weak light flash when all RCIIs are opened for charge separation in dark regulated state. After a series of increasing excitation pulses, the ChlF yield eventually reaches a maximal value F_m_ when RCIIs are all closed. By parameterizing the fluorescence-light response curve of ChlF yield from F_o_ to F_m_, the effective absorption cross section of PSII (σ_PSII_) can be derived. The ChlF yield is controlled by the competition among the processes of fluorescence (*f*), heat dissipation (*h*), and photochemistry (*p*). If C is the scale factor, and K is the rate constant of these processes (Kolber et al., 1998; Xie et al., 2018), then, F_o_ = CK_*f*_ /(K_*f*_+K_*h*_+K_*p*_); F_m_ = CK_*f*_ /(K_*f*_+K_*h*_). In light regulated state, K_*h*_ is assumed to change due to increased activity of non-photochemical quenching (NPQ). F′ = CK_*f*_ /(K_*f*_+*x*K_*h*_+K_*p*_); ${\text{F}}_{\text{m}}^{\prime }$ = CK_*f*_ /(K_*f*_+*x*K_*h*_). In this way, we determined the fluorescence yields F_o_ and F_m_ for dark-regulated state and F' and ${\text{F}}_{\text{m}}^{\prime }$ for light-regulated state according to the biophysical model of Kolber et al. (1998). F_v_ and ${\text{F}}_{\text{q}}^{\prime }$ were calculated, respectively, as:

(2) $$\begin{array}{r} {\text{F}}_{\text{v}}={\text{F}}_{\text{m}}-{\text{F}}_{\text{o}} \end{array}$$

(3) $$\begin{array}{r} {\text{F}}_{\text{q}}^{{\;}^{\prime }\;}={\text{F}}_{\text{m}}^{{\;}^{\prime }\;}-{\text{F}}^{{\;}^{\prime }\;} \end{array}$$

In dark-adapted state, the maximum quantum efficiency of PSII was calculated using the ratio of F_v_/F_m_ as per Kitajima and Butler (1975):

(4) $$\begin{array}{r} {\text{F}}_{\text{v}}/{\text{F}}_{\text{m}}=\left({\text{F}}_{\text{m}}-{\text{F}}_{\text{o}}\right)/{\text{F}}_{\text{m}} \end{array}$$

Rather, the quantum efficiency of photochemical energy conversion in PSII under the light-regulated state, ${\text{F}}_{\text{q}}^{\prime }$/${\text{F}}_{\text{m}}^{\prime }$ ($\Phi_{\text{PSI}{\text{I}}^{\text{′}}}$), was derived as follows (Oxborough et al., 2000):

(5) $$\text{F}\prime_{\text{q}}/\text{F}\prime_{\text{m}}=\left(\text{F}\prime_{\text{m}}-\text{F}\prime \right)/\text{F}\prime_{\text{m}}$$

F_o_' was estimated as (Oxborough and Baker, 1997):

(6) $$\begin{array}{r} {\text{F}}_{\text{o}}^{{\;}^{\prime }\;}={\text{F}}_{\text{o}}/\left({\text{F}}_{\text{v}}/{\text{F}}_{\text{m}}+{\text{F}}_{\text{o}}/{\text{F}}_{\text{m}}^{{\;}^{\prime }\;}\right) \end{array}$$

The photochemical quenching of variable fluorescence (${\text{F}}_{\text{q}}^{\prime }$/${\text{F}}_{\text{v}}^{\prime }$), which quantifies the fraction of functional RCII (Q_A_ oxidized) at each light level in the open state, was calculated as Machlis (1963):

(7) $${\text{F}}_{\text{ q}}^{\prime }/{\text{F}}_{\text{ v}}^{\prime }=\left({\text{F}}_{\text{ m}}^{\prime }-{\text{F}}^{\prime }\right)/\left({\text{F}}_{\text{ m}}^{\prime }-{\text{F}}_{\text{ o}}^{\prime }\right)$$

ETR_RCII_ (mol e^−^ mol RCII^−1^ s^−1^) in functional RCII was derived as the product of PAR (E, μmol quanta m^−2^ s^−1^), the σ_PSII_ at E (Å RCII^−1^) and the efficiency with which charge separation occurs in RCII. The constant value 6.022 × 10^−3^ converts μmol quanta to quanta and Å^2^ (10^−20^ m^2^) to m^2^ (Kolber and Falkowski, 1993).

(8) $$\begin{array}{r} {\text{ETR}}_{\text{RCII}}=\text{E}\times \sigma_{\text{PSII}}^{\text{′}}\times \frac{{\text{F}}_{\text{q}}^{\text{′}}}{{\text{F}}_{\text{v}}^{\text{′}}}\times 6.022\times 10^{-3} \end{array}$$

The NPQ at given light level was calculated as the normalized Stern-Volmer quenching coefficient, defined as NPQ_NSV_ (Mitchell et al., 2002):

(9) $$\begin{array}{l} {\text{NPQ}}_{\text{NSV}={\text{F}}_{\text{o}}^{{\;}^{\prime }\;}/{\text{F}}_{\text{v}}^{{\;}^{\prime }\;}}\\ \;\;\;\;=({\text{F}}_{\text{o}}/({\text{F}}_{\text{v}}/{\text{F}}_{\text{m}}+{\text{F}}_{\text{o}}/{\text{F}}_{\text{m}}^{{\;}^{\prime }\;}))/({\text{F}}_{\text{m}}^{{\;}^{\prime }\;}-{\text{F}}_{\text{o}}/({\text{F}}_{\text{v}}/{\text{F}}_{\text{m}}+{\text{F}}_{\text{o}}/{\text{F}}_{\text{m}}^{{\;}^{\prime }\;})) \end{array}$$

### Carbon Fixation of ^14^C Assimilation

^14^C-uptake incubation experiments were conducted with water collected from the surface (~5 m) and from depths corresponding to 50, 30, 10, and 1% of surface PAR. The sampling depths were determined according to the estimated *K*_d_ (Equation 1). To simulate submarine irradiances, we screened sunlight by different combinations of neutral density filters. Seawater samples were prescreened through 200 μm mesh and then placed in two light and one dark acid-cleaned polycarbonate bottles of 250 mL. Carbon fixation was obtained from the uptake of NaH^14^CO_3_ (Strickland and Parsons, 1972), which was filled into each incubation bottle with trace amounts (10 μCi). The incubators were maintained at *in situ* temperature by a seawater circulation system. After 6 h incubation, water samples were filtered through 25 mm GF/F filters under low vacuum (<0.04 MPa). Radioactivity on the filters was measured with a liquid scintillation counter (Tri-Carb 2900TR) after removing residual inorganic carbon by concentrated HCl fuming overnight and immersing the filters within scintillation counting cocktail (10 mL; Ultima Gold, PerkinElmer). Carbon uptake rates (*PP*_z,t_, mg C (mg chla)^−1^ h^−1^ m^−3^) derived from 6 h incubations were calculated as follows:

(10) $$\begin{array}{r} PP\;\text{z, t}=\frac{1.05\times p\left(C\right)\left(R_{\text{a}}-R_{d}\right)}{R\times T\times p\left(Chla\right)} \end{array}$$

Where *R*_a_ is the average activity of NaH^14^CO_3_ added to three light bottles (kBq), *R*_d_ is the activity of NaH^14^CO_3_ added to dark bottle (kBq), *T*, and *p*(*Chla*) are the incubation time (h) and total Chl *a* concentration (mg m^−3^), respectively. *p(C)* is the total amounts of CO_2_ concentration (mg m^−3^), which was estimated from salinity as per an empirical equation *p(C)* = (0.067 × Salinity−0.05) × 12,000. *R* is the total activity of NaH^14^CO_3_ added to the incubation bottle (kBq). The isotope discrimination between ^14^C and ^12^C was corrected by the factor of 1.05.

The integrated *PP*_z,t_ over Z_eu_ depth [*PP*_zeu_, mg C (mg chla)^−1^ h^−1^ m^−2^] was calculated as:

(11) $$PP_{\text{Zeu}}=\sum_{n}^{n+1}\frac{\left(PP_{i}+PP_{i+1}\right)}{2}\times (D_{i+1}-D_{i})$$

where *PP*_*i*_ is the *PP*_z,t_ at sampling layer *i* [mg C (mg chla)^−1^ h^−1^ m^−3^], *n* is the number of sampling layer, and *D*_*i*_ is the depth at sampling layer *i* (m).

### Model Assumption and FRRF-Based Carbon Fixation

The conversion factor Φ_e : C_/*n*_PSII_ between the ETR_RCII_ (mol e^−^ mol RCII^−1^ s^−1^) and FRRF-measured carbon fixation (mol C mol chl *a*^−1^ s^−1^) was calculated as:

(12) $$\begin{array}{l} \Phi_{\text{e}:\text{C}}/n{\;}_{\text{PSII}}\left(\frac{\text{mol }{\text{e}}^{-}\text{mol chl }a}{\text{mol C mol RCII}}\right)\\ =\frac{{\text{ETR}}_{\text{RCII}}(\text{mol }{\text{e}}^{-}\text{mol }{\text{RCII}}^{-\text{1}}{\text{s}}^{-\text{1}})}{\text{Carbon fixation }(\text{mol C mol chl }a^{-\text{1}}\;{\text{s}}^{-\text{1}})} \end{array}$$

Although the Φ_e : C_/*n*_PSII_ has provided a potential basis for improving estimates of phytoplankton primary productivity, the magnitude of Φ_e : C_/*n*_PSII_ is well-known to change significantly (1.15–54.2) with a multitude of interacting environmental factors (Boyd et al., 1997). The statistical error remains larger even if a constant Φ_e : C_/*n*_PSII_ derived from the averaging has been used in previous field studies (Schuback et al., 2016; Zhu et al., 2017). Therefore, we could not assume a permanent value for Φ_e : C_/*n*_PSII_ to estimate the *GP*_z,t_ of natural phytoplankton in field experiment. Schuback et al. (2015) found a strong correlation between the expression of NPQ_NSV_ and Φ_e : C_/*n*_PSII_ (*R*^2^ = 0.70, *P* < 0.0001), subsequently presented that the use of NPQ_NSV_ can help to predict ETR_RCII_ required Φ_e : C_/*n*_PSII_ and FRRF-derived carbon fixation without the need for any additional measurements and inherent assumptions, since ETR_RCII_ estimate is tightly paired with corresponding NPQ_NSV_ estimate. Actually, such abiotic and biotic factors would be lost using a static (regional) Φ_e : C_/*n*_PSII_ especially to monitor the physiological responses to ambient changes on primary productivity, but are desirably captured with the NPQ_NSV_-based approach. As such, the NPQ_NSV_-based Φ_e : C_/*n*_PSII_ approach is realistic and crucial if the aim is to monitor the effects of environmental variations on primary productivity of natural phytoplankton assemblages. Meanwhile, this approach is not labor-intensive and practical for routine field sampling over large spatial scales. The calculation equation for NPQ_NSV_-based Φ_e : C_/*n*_PSII_ was shown as follows (Schuback et al., 2015):

(13) $$\text{ ​​}\Phi \text{​​}{\;}_{\text{e:C}}/n_{\text{PSII}}\;=8792.4\;{\text{NPQ}}_{\text{NSV}}-733.21\;{\text{NPQ}}_{\text{NSV}}^{2}-1477.1$$

Thereupon we proposed a hypothesis for FRRF-derived carbon fixation (*F*_C_) without the need for additional Φ_e : C_/*n*_PSII_ in natural phytoplankton assemblages. The relationship between NPQ_NSV_ and *F*_C_ according to the above Equations (7, 8, 12, and 13) was calculated as:

(14) $$F_{\text{C}}=\frac{\text{E}\times \sigma_{\text{PSII}}^{\prime }\times \frac{\left({\text{F}}_{\text{m}}^{\prime }-{\text{F}}^{\prime }\right)}{\left({\text{F}}_{\text{m}}^{\prime }-{\text{F}}_{\text{o}}^{\prime }\right)}\times 6.022\times 10^{-3}}{8792.4\;{\text{NPQ}}_{\text{NSV}}-733.21\;{\text{NPQ}}_{\text{NSV}}^{2}-1477.1}$$

Where *F*_C_ is the FRRF-measured gross carbon fixation per unit Chl *a* (mol C mol chl *a*^−1^ s^−1^), FRRF-*GP*_z,t_ [mg C (mg chla)^−1^ h^−1^] was calculated as: *GP*_z,t_ = 3.85 × 10^4^ × *F*_C_, the factor 3.85 × 10^4^ converts mol C mol chl *a*^−1^ s^−1^ to mg C (mg chla)^−1^ h^−1^ (Smyth et al., 2004).

### Statistical Analyses

Spearman and Pearson correlation analysis were used to examine covariance of photosynthetic parameters (such as NPQ, F_v_/F_m_, ${\text{F}}_{\text{q}}^{\prime }$/${\text{F}}_{\text{m}}^{\prime }$, ${\text{F}}_{\text{q}}^{\prime }$/${\text{F}}_{\text{v}}^{\prime }$, and ETR_RCII_) with environment factors (SPSS, Version 19, IBM). Regression models and *t*-test were then applied for testing significant differences between groups of data. However, these regression models provided the predictive shape of the response curve of the photosynthetic parameters to environmental variables and highlighted the variance (*R*^2^).

## Results

### NPQ_NSV_

**Figure 3:**
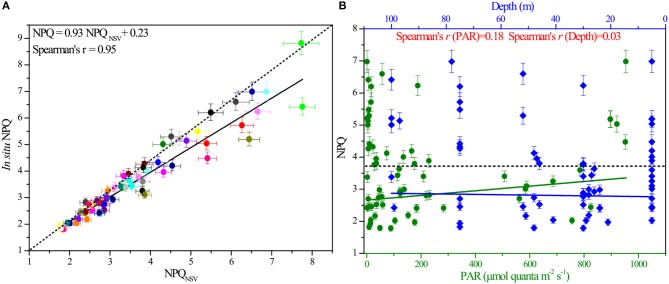
The correlation between *in situ* NPQ and NPQ_NSV_, as well as the responses of *in situ* NPQ to light and depth. **(A)** *In situ* NPQ derived from FRRF measurements plotted against NPQ_NSV_ calculated by fluorescence parameters of the same water (Spearman's rank correlation coefficient *S* = 0.95, *n* = 72). **(B)** Responses of NPQ to changes in light and depth over the course of the *in situ* experiment (*S*_PAR_ = 0.18, *S*_depth_ = 0.03). NPQ was derived from the *in situ* FRRF measurements and is unitless. The dashed line indicates the average value of NPQ.

Samples of *in situ* NPQ were similarly taken at 20 stations in the BOB (Figure 1). FRRF-based fluorescence curve fits were retrieved to derive the NPQ_NSV_ at 12 light levels, while *in situ* NPQ data were simultaneously obtained using the FastOcean FRRF3 sensor. The NPQ_NSV_ values were calculated from the relationship presented in Equation (9), estimated as F_o_'/F_v_'. Not surprisingly, *in situ* NPQ values and the above calculated NPQ_NSV_ in the same waters were well-correlated (Pearson correlation coefficient *P* = 0.91 for the close correlation between *in situ* NPQ and NPQ_NSV_, *p* < 0.0001, *n* = 72), in turn, confirming that our fundamental fluorescence parameters (i.e., F_o_, F_m_, F′, and ${\text{F}}_{\text{m}}^{\prime }$) are reasonable and reliable. Empirical supporting evidence is presented. Further, the calculated NPQ_NSV_ values could be converted to *in situ* NPQ using their correlated relationships (*in situ* NPQ = 0.93NPQ_NSV_ + 0.23, Figure 3A).

The non-photochemical variable NPQ at *in situ* irradiance levels ranged from 1.78 to 6.98, with an average value (±standard error, SE) of 3.61 ± 1.36, and showed prominent variability both within and between stations in the BOB. Theoretically, this different expression of NPQ appears to be induced by the effects of excess irradiance pressure on the photosynthetic ETC in PSII. To clearly understand the variable pattern at large spatial scales, all the data points of NPQ against PAR and depth were analyzed as shown in Figure 3B. Operationally, this *in situ* parameter showed a remarkably light and depth-independent response (*P*_PAR_ = 0.01, *P*_depth_ = 0.28; *p* > 0.01, *n* = 72).

### Variability of Photosynthetic Parameters

To better understand the efficiency of photochemistry in PSII and its dynamic response to ambient regime observed over large spatial scales, we examined changes of *in situ* FRRF-derived ChlF parameters (F_v_/F_m_, ${\text{F}}_{\text{q}}^{\prime }$/${\text{F}}_{\text{m}}^{\prime }$, and ${\text{F}}_{\text{q}}^{\prime }$/${\text{F}}_{\text{v}}^{\prime }$) for the in-depth analysis of our data, and all data points (*n* = 72) of these photophysiological parameters against depth and light were analyzed to plot the related fitting curves.

**Figure 4:**
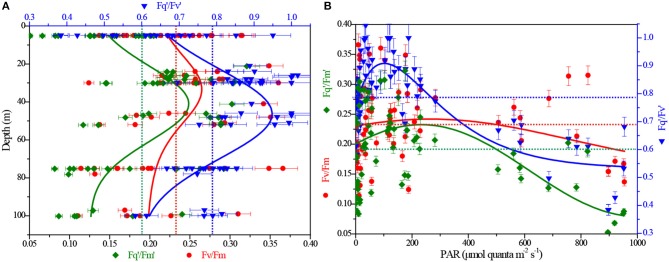
Depth and light dependency of ChlF-derived F_v_/F_m_, ${\text{F}}_{\text{q}}^{\prime }$/${\text{F}}_{\text{m}}^{\prime }$ and ${\text{F}}_{\text{q}}^{\prime }$/${\text{F}}_{\text{v}}^{\prime }$ from FRRF measurements. **(A)** F_v_/F_m_, ${\text{F}}_{\text{q}}^{\prime }$/${\text{F}}_{\text{m}}^{\prime }$ and ${\text{F}}_{\text{q}}^{\prime }$/${\text{F}}_{\text{v}}^{\prime }$ vs. depth; **(B)** F_v_/F_m_, ${\text{F}}_{\text{q}}^{\prime }$/${\text{F}}_{\text{m}}^{\prime }$ and ${\text{F}}_{\text{q}}^{\prime }$/${\text{F}}_{\text{v}}^{\prime }$ vs. light. The red, olive and blue dashed lines represent the averaging of F_v_/F_m_, ${\text{F}}_{\text{q}}^{\prime }$/${\text{F}}_{\text{m}}^{\prime }$ and ${\text{F}}_{\text{q}}^{\prime }$/${\text{F}}_{\text{v}}^{\prime }$, respectively.

Values of F_v_/F_m_, measured in the dark-regulated state, varied from 0.11 to 0.37 and averaged at 0.23 ± 0.04 (±SE). The parameter F_q_'/F_m_', the overall quantum efficiency of photochemical energy conversion in PSII ($\Phi_{\text{PSI}{\text{I}}^{\text{′}}}$) in the light-regulated state, ranged from 0.05 to 0.32, with an average (±SE) of 0.18 ± 0.07. In contrast, ${\text{F}}_{\text{q}}^{\prime }$/${\text{F}}_{\text{v}}^{\prime }$ values [ranged from 0.38 to 1.02, averaged at 0.78 ± 0.15 (±SE)], representing the efficiency of charge separation in functional RCII, were relatively higher at all irradiance levels. This is attributable to the fact that $\Phi_{\text{PSI}{\text{I}}^{\text{′}}}$ served as an estimate of the fraction of open RCII at given light level always approaches one at low irradiance. *In situ* curve fitting, though not statistically significant, variation trends observed for these parameters were similar and representative throughout the BOB. F_v_/F_m_, ${\text{F}}_{\text{q}}^{\prime }$/${\text{F}}_{\text{m}}^{\prime }$, and ${\text{F}}_{\text{q}}^{\prime }$/${\text{F}}_{\text{v}}^{\prime }$ initially increased with depth and displayed a relative maximum at the subsurface (30–50 m), then decreased rapidly down to the minimum nearby the Z_eu_ depth (${\text{R}}_{\text{Depth}}^{2}$ = 0.18, 0.32, and 0.43, respectively; Figure 4A). While at a continuous irradiance of 0~300 μmol quanta m^−2^ s^−1^, they remained high and relatively constant, but declined after continuing to increase in irradiance ($R_{\text{PAR}}^{2}$ = 0.17, 0.32, and 0.77, respectively; Figure 4B).

### ETR_RCII_ and *F*_C_

**Figure 5:**
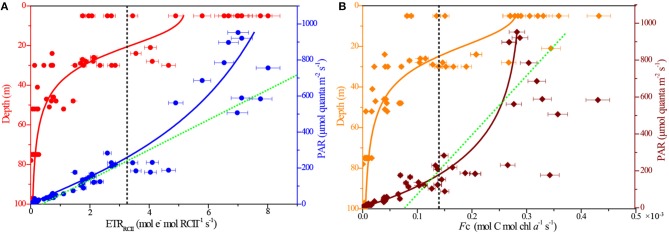
Depth and light responses of rates of ETR_RCII_ and *F*_C_. **(A)** ETR_RCII_ vs. depth (*R*^2^ = 0.68) and PAR (*R*^2^ = 0.87); **(B)** *F*_C_ vs. depth (*R*^2^ = 0.96) and PAR (*R*^2^ = 0.83). The black dashed lines, respectively, indicate their average values. The green dashed lines, respectively, represent the theoretical exponential model of Webb et al. (1974) for the P vs. E curves.

Working with natural phytoplankton assemblages in the BOB, we examined the interacting effects of depth (P vs. D curves) and instantaneous light level (P vs. E curves) on the rates of ETR_RCII_ and *F*_C_ (Figure 5). Both rates were calculated as a function of irradiance and showed the expected light dependency. At broad-scale natural state, however, P vs. E curve was not fit with the exponential model of Webb et al. (1974), who observed all data points would be excluded from the fitting procedure as a result of photoinhibition in lab cultures. Interestingly, the P vs. D and P vs. E curves were simultaneously fit to the logistic model in present study.

The rates of ETR_RCII_ varied greatly, ranging by more than 2 orders of magnitude from 0.01 to 8.01 mol e^−^ mol RCII^−1^ s^−1^, with an average (±SE) of 3.21 ± 0.95 mol e^−^ mol RCII^−1^ s^−1^. Maximum value of *F*_C_ was 0.43 × 10^−3^ mol C mol chl *a*^−1^ s^−1^ at the surface, and the average value (±SE) was 0.14 ± 0.05 × 10^−3^ mol C mol chl *a*^−1^ s^−1^. Very similar responses to depth and irradiance were observed, respectively, between the ETR_RCII_ and *F*_C_ (Figure 5). In our P vs. D curve fits, both ETR_RCII_ and *F*_C_ showed statistically significant decline with depth (*P*_ETR_ = −0.74, *P*_*F*__c_ = −0.75; *p* < 0.0001). In contrast, there was significant increase in the P vs. E curve fits following light addition (*P*_ETR_ = 0.93, *P*_*F*__c_ = 0.87; *p* < 0.0001), indicating the light-dependent responses in both ETR_RCII_ and *F*_C_ for natural phytoplankton were not readily limited by high irradiance under large spatial scales. This result appears to be exemplified to differing degrees by the contrast in the relationship between NPQ and PAR (see Figure 3B). Overall, the P vs. D and P vs. E curves demonstrated significant and interactive effects of depth and irradiance availability on the rates of ETR_RCII_ and *F*_C_.

### Variability of Phytoplankton, FRRF-*GP*_Zeu_ and ^14^C-*PP*_Zeu_

**Figure 6:**
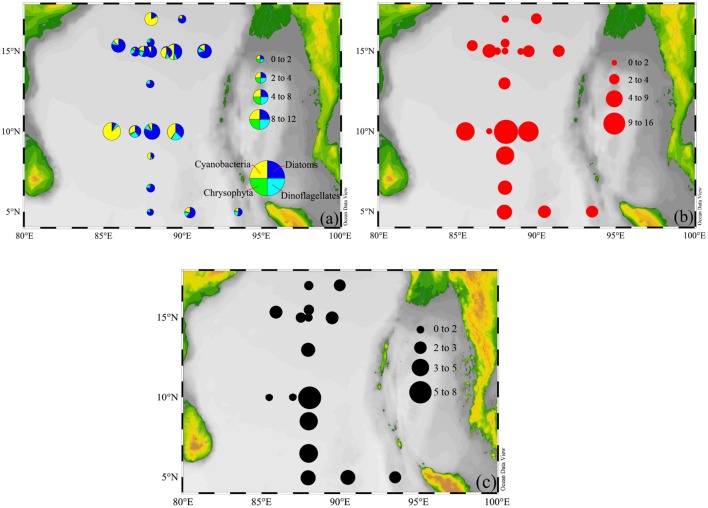
Spatial distributions of **(a)** phytoplankton abundance (×10^4^ cells m^−3^), **(b)** FRRF-*GP*_Zeu_, and **(c)** ^14^C-*PP*_Zeu_ [mg C (mg chla)^−1^ h^−1^ m^−2^] within the upper Z_eu_ depth.

The abundance proportions of phytoplankton classes in the natural community were averaged, consisting of approximately 57% diatoms, 14% dinoflagellates, 26% cyanobacteria, and 3% chrysophytes. The contributions of diatoms and cyanobacteria to total phytoplankton abundance taken together amounted to about 83%, to some extent, further confirming that they were the numerically dominant component of phytoplankton communities, and had crucial role in primary productivity (Figure 6a). The FRRF-*GP*_z,t_ integrated over Z_eu_ depth (*GP*_Zeu_) varied by 2 orders of magnitude across the BOB, from 0.95 to 15.17 mg C (mg chla)^−1^ h^−1^ m^−2^, with an average value (±SE) of 4.62 ± 0.97 mg C (mg chla)^−1^ h^−1^ m^−2^. We observed great changes in the spatial distributions of both phytoplankton abundance and *GP*_Zeu_ across the BOB, however, they did not change in parallel (Figures 6a,b). At all stations (*n* = 20), *GP*_Zeu_ were relatively high at B06, B07, and B09, while the maximum abundance of phytoplankton were at B07, B09, and B12. Even though not statistically significant (*P* = 0.28; *p* > 0.05), the distributional trends observed for phytoplankton abundance and *GP*_Zeu_ were similar throughout the BOB. The high-*GP*_Zeu_ zones were primarily dominated by diatoms and cyanobacteria, where they contributed over more than 78% to the total abundance of phytoplankton. As a consequence, this result further confirms that the variability in diatoms and cyanobacteria appear to be the primary drivers of variability in *GP*_Zeu_.

**Figure 7:**
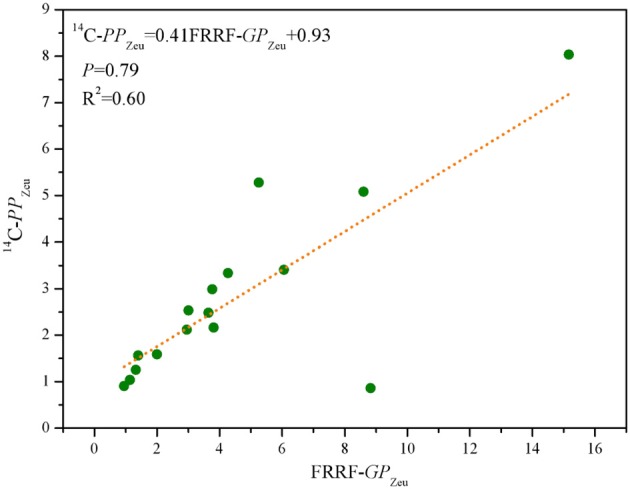
Scatter plots of FRRF-*GP*_Zeu_ and ^14^C-*PP*_Zeu_ [mg C (mg chla)^−1^ h^−1^ m^−2^] for pooled data of 16 stations in the BOB.

^14^C-*PP*_Zeu_ ranged from 0.86 to 8.04 mg C (mg chla)^−1^ h^−1^ m^−2^, with mean (±SE) of 2.79 ± 0.12 mg C (mg chla)^−1^ h^−1^ m^−2^ for 16 stations. As expected, spatial distribution of ^14^C-*PP*_Zeu_ was highly similar with that of FRRF-*GP*_Zeu_ (Figures 6b,c). It is well to emphasize that ^14^C-*PP*_Zeu_ and FRRF-*GP*_Zeu_ across the study area were strongly positively correlated with each other (Figure 7; *P* = 0.79, *R*^2^ = 0.60; *S* = 0.79, *p* < 0.0001, *n* = 16), revealing that our hypothesis (Equation 14) can be reasonably applied to derive FRRF-*GP*_z,t_ rate. As such, we proposed a simple field model for FRRF-carbon estimate in the BOB, without the need for additional Φ_e : C_/*n*_PSII_, which was calculated as per the hypothesis (Equation 14) and the linear *PP*_Zeu_-*GP*_Zeu_ relationship.

(15) $$\text{FRRF-Carbon}=\frac{\text{E}\times \sigma_{\text{PSII}}^{\prime }\times \frac{\left({\text{F}}_{\text{m}}^{\prime }-{\text{F}}^{\prime }\right)}{\left({\text{F}}_{\text{m}}^{\prime }-{\text{F}}_{\text{o}}^{\prime }\right)}}{92.6\;{\text{NPQ}}_{\text{NSV}}-7.7\;{\text{NPQ}}_{\text{NSV}}^{2}-15.6}+0.93$$

Overall, our independent field model is realistic and robust for the FRRF-derived carbon estimate in the BOB, but just our research is not yet fine enough to fully prove its reliability in other oceans. Therefore, more data are needed to further enhance the applicability of FRRF-based field model to other ecosystems.

## Discussion

### Light and Depth Responses of NPQ

As a result observed in Figure 4, the expression of NPQ showed a light and depth-independent response. Although Schuback et al. (2015) revealed that NPQ_NSV_ increased with increasing light and decreased in response to iron addition, she attributed this effect to a more stable irradiance level in incubation experiments, relative to *in situ* flexible light environment. Regardless of irradiance influence, mono-specific laboratory culture of marine phytoplankton species, isolating natural phytoplankton assemblages, may especially contribute to the light and iron dependency of NPQ_NSV_, yet it is unlikely to occur in natural mixed phytoplankton assemblages under open ocean condition, in part because the species-specific differences observed in incubation bottles are not consistent with changes in natural phytoplankton composition observed in field experiments. Furthermore, the *in situ* environmental factors, for example physical and biological instability, variable stratification, temperature and other micronutrients (e.g., Mn, Cu, and Zn) may also slightly affect the conversion of light energy and therefore the expression of NPQ_NSV_ (Georgieva and Yordanov, 1994; Raven et al., 1999; Smyth et al., 2004). In summary, on large spatial scales, it is tempting to speculate that the variation of NPQ could not be simply explained by light and iron-dependent responses, but also needed to be combined with the joint effects of complex taxonomic composition and variable environmental conditions.

### Application of NPQ_NSV_-based Φ_e : C_/*N*_PSII_ for Our Field Model

The NPQ_NSV_ and Φ_e : C_/*n*_PSII_ are not entirely independent parameters. In particular, the process acting to regulate electron transport and process preventing over-reduction of the ETC after charge separation are both controlled by excess excitation energy, it is reasonable to expect that their magnitude mechanistically correlates (Schuback et al., 2016). In addition, a wide variety of endogenous and exogenous mechanisms related to the relaxation of high excitation pressure experienced by the ETC can simultaneously influence NPQ_NSV_ and Φ_e : C_/*n*_PSII_ in a consistent manner (Ruban et al., 2012). From a photophysiological point of view, because excess light energy can be dissipated as heat before reaching RCII, the effects of increased excitation pressure on the ETC will ultimately cause the increased decoupling of CO_2_-assimilation and ETR_RCII_ (Schuback et al., 2015). For instance, the expression of NPQ_NSV_ will increase if light is saturating, and concomitant with an increase in Φ_e : C_/*n*_PSII_ (Kaiblinger and Dokulil, 2006), this is because excess energy transfer to RCII and over-reduction of the ETC can be initially alleviated by a number of alternative electron pathways after charge separation, thereafter resulting in a strong correlation between the NPQ_NSV_ and Φ_e : C_/*n*_PSII_ in PSII (Laureau et al., 2013). For this reason, the applicability of NPQ_NSV_-based Φ_e : C_/*n*_PSII_ is currently crucial to considering in the development of ChlF-based *GP*_z,t_ estimates.

The intimate NPQ_NSV_-Φ_e : C_/*n*_PSII_ relationship in Equation (13) was in some a result of their co-dependence on the ChlF parameters ${\text{F}}_{\text{m}}^{\prime }$, ${\text{F}}_{0}^{\prime }$, and F′. However, these ChlF signals normalized to the rates of ETR_RCII_ are derived by an iterative non-linear fitting procedure (Kolber et al., 1998), indicating that the hypothesis which we proposed in Equation (14) for *F*_C_ from natural phytoplankton assemblages was empirical rather than mechanistic, thereby providing a measure of *GP*_z,t_. Notwithstanding some potential sources of uncertainty in the absolute value of NPQ_NSV_-based Φ_e : C_/*n*_PSII_, the good agreement between our FRRF-derived *GP*_Zeu_ and ^14^C-uptake *PP*_Zeu_ (Figure 7; *P* = 0.79, *R*^2^ = 0.60; *p* < 0.0001) suggests that our independent field model for FRRF-derived primary productivity is operationally robust and suitable in the BOB. Recently, some work have pointed that a close link exist between alternative electron sinks involving midstream plastoquinol oxidase (PTOX) and the expression of NPQ_NSV_, providing a new mechanistic insight into the process on the coupling between Φ_e : C_/*n*_PSII_ and NPQ_NSV_ (Laureau et al., 2013; Alric and Johnson, 2017). Consequently, to further enhance the accuracy and suitability of our FRRF-based field model in the BOB, even in other marine ecosystems, more data are needed in future work.

### Interacting Effects of Ambient Conditions on FRRF Parameters

F_v_/F_m_ has been used to characterize variation in the quantum efficiency of PSII. The maximum value equals 0.65 when all functional RCII are operating at maximum efficiency. Most often, F_v_/F_m_ ranges from 0.65 in highly-productive regions to <0.3 in oligotrophic gyres (Falkowski and Kolber, 1995; Behrenfeld et al., 1996; Jin et al., 2016). In the BOB, most observed values of F_v_/F_m_ (0.11~0.37) were only about half of the values expected for nutrient replete phytoplankton (Figure 4), hence indicating a biophysical consequences of nutrient limitation for phytoplankton assemblages. Consistent with previous observations, the range of DIN and DIP concentrations encountered in the euphotic layer exert a significant influence on values of the F_v_/F_m_ as well as the NPQ_NSV_ and ETR_RCII_ (Table 1). However, nutrient concentrations alone are inadequate to explain and predict the variability of these FRRF parameters over large spatial scales.

**Table 1:** Pearson's rank correlation coefficients between photosynthetic parameters and environmental factors.

**Factors**	**PAR**	**Temperature**	**Salinity**	**DIP**	**DSi**	**DIN**
F_v_/F_m_	−0.127	0.199	−0.156	−0.218	−0.163	−0.182^*^
F_q_'/F_m_'	−0.389^**^	0.237	−0.077	−0.174	−0.184	−0.151
F_q_'/F_v_'	−0.515^**^	0.090	−0.032	0.006	−0.096	−0.032
NPQ_NSV_	0.134	−0.323^*^	0.188	0.282^*^	0.213	0.220
ETR_RCII_	0.929^**^	0.502^**^	−0.352^*^	−0.487^**^	−0.450^**^	−0.459^**^

* Correlation is significant at the 0.05 level;

** *Correlation is significant at the 0.01 level (2-tailed). Values in bold mean that significant correlation is highlighted in this study*.

**Figure 8:**
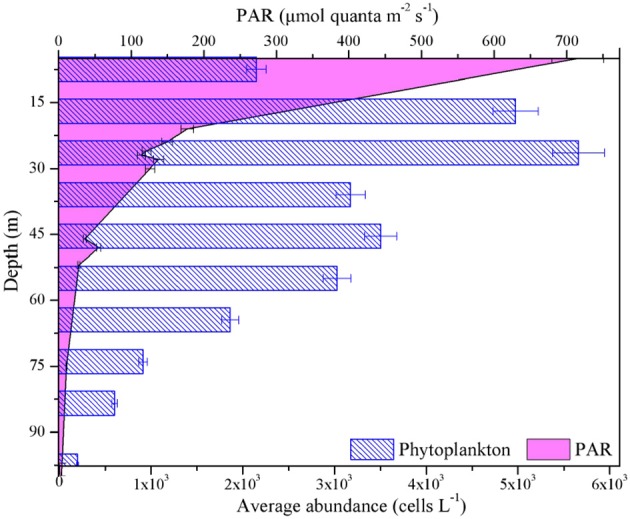
Vertical profiles for average values of phytoplankton abundance and PAR in the BOB.

The average light intensity was comparatively high (~700 ± 338 μmol quanta m^−2^ s^−1^) in surface layer of BOB (Figure 8), F_v_/F_m_, ${\text{F}}_{\text{q}}^{\prime }$/${\text{F}}_{\text{m}}^{\prime }$, and ${\text{F}}_{\text{q}}^{\prime }$/${\text{F}}_{\text{v}}^{\prime }$ concurrently showed an inverse relationship to light availability during high excitation pressure (PAR > 300 μmol quanta m^−2^ s^−1^, Figure 4B and Table 1), thereby indicating that the low values of them in the high-light region near the surface may be caused by the photo-protective mechanisms (including endogenous changes in metabolic energy allocation), some extent, namely the NPQ process (Öquist et al., 1992; Campbell and Tyystjärvi, 2012). Their increase at subsurface further confirms the inhibition of surface supersaturating irradiance and the presence of slow-relaxing NPQ components (Figure 4A). Likewise, low temperature usually has limited the ETR_RCII_ due to low ribulose-1, 5-bisphosphate carboxylase/oxygenase activity (van de Poll and Buma, 2009; Jin et al., 2016). Accordingly, the negative correlation between these ChlF parameters and nutrients is likely attributable to the changes of temperature and light environment in vertical profiles (Figure 8 and Table 1).

Furthermore, average abundance of phytoplankton responded strongly to depth in the BOB, similarly with maximum value near the subsurface (Figure 8). Therefore, the phytoplankton may also affect these *in situ* photophysiological parameters, as demonstrated by rapid changes in relative flash output and wavelengths, as well as the expression of NPQ. Indeed, the variability in F_v_/F_m_, ${\text{F}}_{\text{q}}^{\prime }$/${\text{F}}_{\text{m}}^{\prime }$, and ${\text{F}}_{\text{q}}^{\prime }$/${\text{F}}_{\text{v}}^{\prime }$ is thought to be highly associated with physiological state of the phytoplankton assemblages (Olaizola et al., 1996). Recently, a number of studies have collectively shown that change in community structure within phytoplankton assemblages appears to be a factor increasingly important in explaining patterns of photosynthetic parameters, likely reflecting the selection of better adapted species by environmental drivers (Zhu et al., 2017; Kulk et al., 2018; Xie et al., 2018). It is well to emphasize that diatoms and cyanobacteria have made a significant contribution to the dynamics of these photosynthetic parameters in this study. In summary, the changes in F_v_/F_m_, ${\text{F}}_{\text{q}}^{\prime }$/${\text{F}}_{\text{m}}^{\prime }$, and ${\text{F}}_{\text{q}}^{\prime }$/${\text{F}}_{\text{v}}^{\prime }$ associated with depth may have resulted from both, photophysiological responses to ambient conditions and changes in community structure of phytoplankton assemblages.

Ideally, changes in ETR_RCII_ and *F*_C_ in phytoplankton field assemblages were limited by excess irradiance (Webb et al., 1974; Mitchell et al., 2002). However, on large spatial scales, these two rates of variability were, to a large extent, not susceptible to light fluctuations in ambient light of sufficient intensity (Figure 5). Since environmental forcing generates selective pressures on phytoplankton community structure presenting within an ecosystem, resulting in marked changes in photosynthetic parameters (as discussed above). Taxonomic shifts of phytoplankton community facilitate the selection of better adapted species to optimize photosynthetic efficiency under any particular set of ambient light condition (Figures 6, 8). Furthermore, high excitation pressure will effectively select for phytoplankton assemblages with the best ability to adapt for high irradiance condition by adjusting effective absorption cross section of PSII (Jin et al., 2016) and ameliorating the flow of excitation energy into of PSII (Schuback et al., 2015), potentially leading to the high values of ETR_RCII_ and *F*_C_ under high irradiances.

### Contrasting Primary Productivity Between FRRF Models and ^14^C Dataset

To verify whether the NPQ_NSV_-proxy hypothesis is possible to apply in the BOB, we subsequently compared our model with previously reported models from other ecosystems and synchronously measured *PP*_z,t_ (^14^C) dataset. Apart from the calculation of *GP*_z,t_ from the field model described in present study, another simple approach (defined as Model 1) also involved measuring the instantaneous depth-dependent rates of *GP*_z,t_, which when integrated over Z_eu_ depth produced the values of *GP*_Zeu_ (Smyth et al., 2004). The factor of 1.56 × 10^−4^ accounting for the conversion from mol C mol chl *a*^−1^ s^−1^ to mg C (mg chla)^−1^ h^−1^ includes the following conversions: 12 g C mol^−1^ C, 892 g Chl *a* mol^−1^, 3,600 s h^−1^, 6.02 × 10^23^ molecules mol^−1^ and 10^20^ m^−2^ photon.

(16) $$GP{\;}_{\text{z,t}}=1.56\times 10^{-4}\Phi_{\text{PSII}\prime }\sigma \prime_{\text{PSII}}\text{E Chl }a$$

In this model, the *GP*_z,t_ is calculated as the product of the concentration of per RCII in PSII, the effective cross-section of RCII (σ_PSII_), the quantum efficiency of photosynthesis ($\Phi_{\text{PSI}{\text{I}}^{\text{′}}}$) and irradiance (E).

(17) $$GP_{\text{Zeu}}=\sum_{n}^{n+1}\frac{\left(GP_{i}+GP_{i+1}\right)}{2}\times (D_{i+1}-D_{i})$$

Where *GP*_*i*_ is the *GP*_z,t_ at sampling layer *i, n* is the number of sampling layer, and *D*_*i*_ is the depth at sampling layer *i*.

Smyth et al. (2004) particularly proposed the $\Phi_{\text{PSI}{\text{I}}^{\text{′}}}$ already included the effect of NPQ_NSV_ which could be manifested by a reduction in σ_PSII_ from its maximum value. The measurements of changes in σ_PSII_ under a variety of background irradiance reveal that the effect of NPQ_NSV_ on the σ_PSII_ is relatively small (0.15–0.20 σ_PSII_ change per unit of NPQ_NSV_ change) (Falkowski et al., 1986). However, this use of $\Phi_{\text{PSI}{\text{I}}^{\text{′}}}$ relative to FRRF-derived NPQ_NSV_ data could lead to an overestimation of the *GP*_z,t_ (see below).

Theoretically, four electrons derived from water are subsequently used to reduce a single molecule of CO_2_ to the level of carbohydrate. Nonetheless, energy losses occur primarily during the processes of excitation energy transfer from the light-harvesting antenna pigments to the RCII, moreover, not all electrons from RCII are further transferred to the terminal acceptors of PSI, such as CO_2_, some are used to reduce ${\text{NO}}_{3}^{-}$ and ${\text{SO}}_{4}^{2-}$ (Kolber and Falkowski, 1993). Electron flow between water and terminal acceptors is coupled in steady state, and the photosynthetic quotients (PQs) for new production are estimated to be 1.1–1.4, even 1.8 or higher that appears to result from comparisons of gross oxygen production to net CO_2_ assimilation (Laws, 1991). It is well to emphasize that the photosynthetic rates from the FRRF (gross) for natural phytoplankton communities will approach the theoretical maximum (*GP*_max_), especially if we assume that the PQ is 1. The *GP*_max_ was calculated on the basis of PSII charge separation rate per unit volume (*JV*_PSII_, electrons (PSII m^−3^) s^−1^), which generally correlates well with the photosynthetic O_2_ evolution (Kolber and Falkowski, 1993; Hoppe et al., 2015).

(18) $$\begin{array}{l} JV_{\;\text{PSII}}=\sigma_{\text{PSII}}\prime \times \;[\text{RCII}]\;\times \;(1\text{-C})\times \text{E}\\ \;\;=\frac{\left({\text{F}}_{\text{m}}\times {\text{F}}_{\text{0}}\right)}{\left({\text{F}}_{\text{m}}-{\text{F}}_{0}\right)}\times \Phi_{{\text{PSII}}^{\prime }}\times \frac{{\text{K}}_{\text{R}}}{{\text{E}}_{\text{LED}}}\times \text{E} \end{array}$$

Where [RCII] is the concentration of functional PSII reaction centers, 1-C is the fraction of RCII in the open state (QA oxidized, capable of stable charge separation), E_LED_ is the measuring beam intensity with units of photons m^−2^ s^−1^, K_R_ is an instrument-specific constant, with units of photons m^−3^ s^−1^. Consequently, the *JV*_PSII_ roughly provides an estimate of *GP*_max_ with units of mg C (mg chla)^−1^ h^−1^ (defined as Model 2). The constant *k* includes the following conversions: 3,600 s h^−1^ and 0.25 C quanta^−1^.

(19) $$\begin{array}{r} {GP}_{max}=k\times {JV}_{\text{PSII}} \end{array}$$

**Figure 9:**
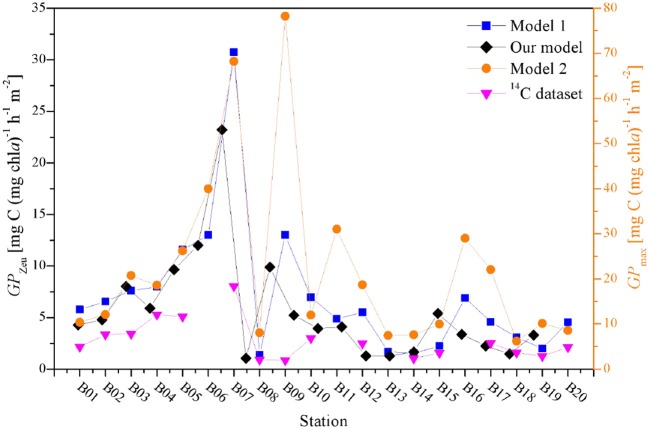
A comparison among the water column integrated primary production from different FRRF approaches and synchronized ^14^C dataset.

Therefore, a comparison of the water column integrated primary production is obtained from the instantaneous FRRF models and synchronized ^14^C dataset (Figure 9). Although the dataset was limited, changes in absolute values of these models among sampling stations were statistically significant (*S* > 0.79, *p* < 0.0001), suggesting that the rates of primary productivity derived from our independent field model can be acquired accurately and reasonably. The operational efficiency of photosynthesis has been estimated from the ratio of *GP*_Zeu_ and *GP*_max_ to be averagely about 24% for natural phytoplankton assemblages. Although estimates of the theoretical upper limit of photosynthetic efficiency in microalgae have not been conducted as systematically, this average efficiency is more double than the theoretical efficiency of plant photosynthesis (Ort et al., 2011). Meanwhile, Ort et al. (2011) proposed that the primary reason why the observed photosynthetic efficiency in field experiment is higher than theoretical efficiency is light adaptation of photosynthesis. Indeed, over large spatial scales, the photosynthesis of phytoplankton assemblages in the BOB responds non-linearly to increases in insolation (Figures 4,5).

## Ethics Statement

I would like to declare on behalf of my co-authors that the described work applies to our study and is original research that has not been published previously, and not under consideration for publication elsewhere, in whole or in part.

## Author Contributions

JS conceived the ideas and designed methodology. YW and XZ performed the experiments and analysis. YW wrote the manuscript and prepared the tables and figures. HL provided the data of synchronous ^14^C-assimilation. All authors edited the manuscript. No conflict of interest exits in the submission of this manuscript, and manuscript is finally approved by all authors for publication.

### Conflict of Interest Statement

The authors declare that the research was conducted in the absence of any commercial or financial relationships that could be construed as a potential conflict of interest.
